# Genetic diversity and phylogenetic relationships in local cattle breeds of Senegal based on autosomal microsatellite markers

**DOI:** 10.14202/vetworld.2015.994-1005

**Published:** 2015-08-18

**Authors:** Ndèye Penda Ndiaye, Adama Sow, Guiguigbaza-Kossigan Dayo, Saliou Ndiaye, Germain Jerôme Sawadogo, Mbacké Sembène

**Affiliations:** 1 Department of Animal Biology, FST (UCAD), Dakar Fann-PO 5005, Laboratory of Endocrinology and Radio-immunology, EISMV, Dakar Fann-PO 5077, Senegal; 2 Laboratory of Endocrinology and Radio-immunology, EISMV, Dakar Fann-PO 5077, Senegal; 3 CIRDES, Bobo Dioulasso 01-PO 454, Burkina Faso; 4 ENSA, University of Thiès, Thiès RP-PO A 296; 5 Laboratory of Endocrinology and Radio-immunology, EISMV, Dakar Fann-PO 5077, Senegal; 6 Department of Animal Biology, FST (UCAD), Dakar Fann-PO 5005, Laboratory CBGP, IRD, Dakar Bel Air- PO 1386, Senegal

**Keywords:** cattle, genetic diversity, microsatellite markers, phylogenetic analysis, Senegal

## Abstract

**Aim:**

In Senegal, uncontrolled cross-breeding of cattle breeds and changes in production systems are assumed to lead to an increase of gene flow between populations. This might constitute a relevant threat to livestock improvement. Therewith, this study was carried out to assess the current genetic diversity and the phylogenetic relationships of the four native Senegalese cattle breeds (Gobra zebu, Maure zebu, Djakoré, and N’Dama).

**Methods:**

Genomic DNA was isolated from blood samples of 120 unrelated animals collected from three agro-ecological areas of Senegal according to their phenotypic traits. Genotyping was done using 11 specific highly polymorphic microsatellite makers recommended by Food and Agriculture Organization. The basic measures of genetic variation and phylogenetic trees were computed using bioinformatics’ software.

**Results:**

A total of 115 alleles were identified with a number of alleles (Na) at one locus ranging from 6 to 16. All loci were polymorphic with a mean polymorphic information content of 0.76. The mean allelic richness (Rs) lay within the narrow range of 5.14 in N’Dama taurine to 6.10 in Gobra zebu. While, the expected heterozygosity (H_E_) per breed was high in general with an overall mean of 0.76±0.04. Generally, the heterozygote deficiency (F_IS_) of 0.073±0.026 was relatively due to inbreeding among these cattle breeds or the occurrence of population substructure. The high values of allelic and gene diversity showed that Senegalese native cattle breeds represented an important reservoir of genetic variation. The genetic distances and clustering trees concluded that the N’Dama cattle were most distinct among the investigated cattle populations. So, the principal component analyses showed qualitatively that there was an intensive genetic admixture between the Gobra zebu and Maure zebu breeds.

**Conclusions:**

The broad genetic diversity in Senegalese cattle breeds will allow for greater opportunities for improvement of productivity and adaptation relative to global changes. For the development of sustainable breeding and crossbreeding programs of Senegalese local breeds, effective management is needed towards genetic selection and transhumance to ensure their long-term survival.

## Introduction

Senegal is an oceanic country, located in West African continent. Longtime practiced of breeding, livestock keeping represents an important source of livelihood for 3.5 million of people, and contributes to 35% of primary sector gross domestic product (GDP) and 7% of national GDP [1]. Cattle are part of the most important domestic livestock species for local communities. Four local cattle breeds have been distinguished namely: Gobra zebu, Maure zebu, Djakoré and N’Dama Taurine, which are found in different agro-ecological systems, from Sahelan to Soudano-Guinea climate. The Gobra zebu was introduced to Senegal in the second half of the eighth century in the Basin of Fouta Toro [2]. Whereas, the Maure zebus commonly found in Mauritania, Mali, and the Niger loop, are bred along the Mauritania border, more precisely in the Senegal river valley [3]. The Djakoré cattle is medium sized compared to Gobra with a barely marked hump. Based on its phenotypic characteristics and geographical distribution, it is thought to result from natural crossings between Gobra zebu and N’Dama [4]. The Djakoré cattle are supposed to be partly trypanotolerant (tolerant to trypanosomoses, due to *Trypanosoma* sp.) and are spread in the central part of the country, more or less infested with tsetse flies [5]. They are used in as traction power to plow crop farms plugging [6]. The N’Dama taurine also known as “West African Longhorn,” originated from Fouta Djallon (Guinea) [7]. N’Dama cattle are a very rustic and trypanotolerant [8]. There are bred in the southern part which is infested by tsetse flies [9].

In order to achieve food security, Senegal has developed policies for the improvement of livestock production by bovine artificial insemination. Local cattle are continually crossed with exotic breeds [10]. In the other hand, transhumance is widely used in the traditional way of livestock management in West Africa, especially after the severe droughts in the 1970’s and 1980’s [11]. So, transhumance is another obvious way to genetic mixtures between cattle populations from different countries, because of the porosity of borders. In this context, Ndiaye *et al*. [12] has used the cytochrome b gene in order to identify the Senegalese cattle breeds. However, only the Gobra zebu was distinguished amongst the other local breeds (Djakoré and N’Dama) and exotic breeds. This showed that the magnitude of crossings was intensively in the vicinity of these breeds and more particularly in the “Bassin Arachidier” area.

As a result, uncontrolled crossings of cattle breeds and changes in production systems are assumed to lead to a very confused genetic structure of local cattle with increasing gene flow between populations. Breed’s characterization is very important for the assessment of genetic diversity, the conservation of genetic resources [13], and their development in the context of global changes [14].

Gobra zebu, Maure zebu, and N’Dama have been the subject of population genetics analyses among studies conducted on a continental scale to retrace indicine and taurine migration across Africa [15-17]. This study was aimed to assess the genetic diversity and phylogenetic relationships among four local cattle breeds, including the Djakoré breed, which has not been characterized up to now.

## Methods

### Ethical approval

This study was approved by the Ethics Committee of the Cheikh Anta Diop University of Dakar. Signed consent of all participants was obtained after the study was fully explained.

### Animal sampling

**Figure-1:**
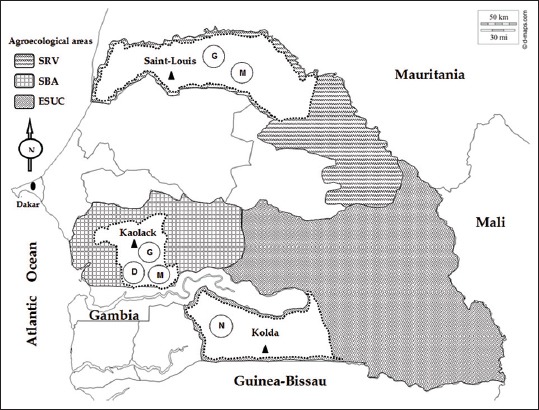
Localization of study sites in three agro-ecological areas of Senegal. Senegal river valley; South of “Bassin Arachidier”; Eastern Senegal and Upper Casamance. Abbreviations of breed names are as follows: D: Djakoré; G: Gobra zebu; M: Maure zebu; N: N’Dama.

Sampling was carried out from October to December 2013 in three agropastoral regions of Senegal namely Saint-Louis (16°02’00’’N and 16°30’00’’W), Kaolack (14°08’35’’N and 16°05’45’’W) and Kolda (13°01’60’’N and 14°52’00’’W). These regions located in three-agro-ecological areas (Figure-1) represent the distribution area of Gobra zebu, Maure zebu, Djakoré and N’Dama breeds. The samples were collected in 15 localities through the study areas and in the Zootechnical Research Center (ZRC) of Kolda. The choice of localities in each region has been done according the availability of the targeted breed. In each geographical area, at least five sites were considered in order to have a representative sample. The selection of breeding stocks was done mainly depending on the ability of breeders to provide the required information in the structured survey questionnaires. A total of 30 farmers and 4 herdsmen of ZRC of Kolda, including 15, 10 and 5 farmers, in the regions of Kaolack, Saint-Louis, and Kolda, were surveyed. For each site, a maximum of 8 herds was surveyed, respectively. In the ZRC of Kolda, 4 reproducer flocks were sampled.

The animals were randomly sampled and classified according to the age of the animal and physiological status of females. To ensure the representativeness of the sample with the least possible relation between animals, a maximum of eight animals was sampled by the herd and four by each reproduction flock. Thus, the samples were composed males subjects aged between 13 and 144 months, and females with age between 18 and 192 months. For each of the four local cattle breed (Gobra zebu, Maure zebu, Djakoré, and N’Dama), 30 individuals were sampled (Table-1). Farmers who participated in the study received free veterinary care for their cattle on the visit date.

**Table-1:** Repartition of sampled subjects per cattle breed according to geographical area (Agro-ecological areas: Senegal river valley (VFS); SBA; ESUC).

Regions and agro-ecological areas	Saint-Louis (SRV)	Kaolack (SBA)	Kolda (ESUC)	Overall
Gobra zebu	13	17	-	30
Maure zebu	26	4	-	30
Djakoré	-	30	-	30
N’Dama	-	-	30	30
Overall	39	51	30	120

SRV=Senegal river valley, SBA=South of Bassin Arachidier, ESUC=Eastern Senegal and upper Casamance

### Blood samples collection

Four milliliters of blood were collected from the jugular vein puncture in vacuum tubes (BD Vacutainer^®^ Systems, Plymouth, United Kingdom) containing EDTA as an anticoagulant and stored at 4°C until further use. During the blood sampling, adequate measures were taken to minimize pain and discomfort.

### Microsatellites amplifications and analysis

DNA was isolated according to standard protocol Gentra Puregene Blood kit designed to extract DNA from whole blood and developed by QIAGEN^®^ group. For genotyping, twelve microsatellites were selected from the panel recommended by the Food and Agriculture Organization and the International Society for Animal Genetics for genetic characterization of cattle breeds [13]. The choice of these markers was mainly effectuated in terms of their technical characteristics (good aptitude to amplification and easy interpretation of typing), and their genetic characteristics (number of alleles, localization and repartition through the genome). For the particular characteristics of these microsatellites loci (Supplement Table-1).

**Supplement Table-1:** Characteristics of microsatellites markers included in this study.

Locus^a^	Chromosome number	Primer name	Primer sequences (5’→3’) Forward/Reverse/Forward FM13^b^	Annealing temperature (C°)
INRA063 (D18S5)	18	INRA063F INRA063R INRA063FM13	ATTTGCACAAGCTAAATCTAACC AAACCACAGAAATGCTTGGAAG CACGACGTTGTAAAACGACATTTGCACAAGCTAAATCTAACC	55
INRA037 (D10S12)	10	INRA037F INRA037R INRA037FM13	GATCCTGCTTATATTTAACCAC AAAATTCCATGGAGAGAGAAAC CACGACGTTGTAAAACGACGATCCTGCTTATATTTAACCAC	50
MM12 (D9S20)	9	MM12F MM12R MM12FM13	CAAGACAGGTGTTTCAATCT ATCGACTCTGGGGATGATGT CACGACGTTGTAAAACGACCAAGACAGGTGTTTCAATCT	55
HEL9 (D8S4)	8	HEL9F HEL9R HEL9FM13	CCCATTCAGTCTTCAGAGGT CACATCCATGTTCTCACCAC CACGACGTTGTAAAACGACCCCATTCAGTCTTCAGAGGT	60
HEL1 (D15S10)	15	HEL1F HEL1R HEL1FM13	CAACAGCTATTTAACAAGGA AGGCTACAGTCCATGGGATT CACGACGTTGTAAAACGACCAACAGCTATTTAACAAGGA	55
ETH10 (D5S3)	5	ETH10F ETH101R ETH10FM13	GTTCAGGACTGGCCCTGCTAACA CCTCCAGCCCACTTTCTCTTCTC CACGACGTTGTAAAACGAC GTTCAGGACTGGCCCTGCTAACA	60
ETH152 (D5S1)	5	ETH152F ETH152R ETH152FM13	TACTCGTAGGGCAGGCTGCCTG GAGACCTCAGGGTTGGTGATCAG CACGACGTTGTAAAACGACTACTCGTAGGGCAGGCTGCCTG	55
BM1818 (D23S21)	23	BM1818F BM1818R BM1818FM13	AGCTGGGAATATAACCAAAGG AGTGCTTTCAAGGTCCATGC CACGACGTTGTAAAACGAC AGCTGGGAATATAACCAAAGG	55
BM2113 (D2S26)	2	BM2113F BM2113R BM2113FM13	GCTGCCTTCTACCAAATACCC CTTCCTGAGAGAAGCAACACC CACGACGTTGTAAAACGACAGCTGCCTTCTACCAAATACCC	55
ETH225 (D9S1)	9	ETH225F ETH225R ETH225FM13	GATCACCTTGCCACTATTTCCT ACATGACAGCCAGCTGCTACT CACGACGTTGTAAAACGACGATCACCTTGCCACTATTTCCT	55
TGLA53 (D16S3)	16	TGLA53F TGLA53R TGLA53FM	GCTTTCAGAAATAGTTTGCATTCA ATCTTCACATGATATTACAGCAGA CACGACGTTGTAAAACGACGCTTTCAGAAATAGTTTGCATTCA	55
TGLA122 (D21S6)	21	TGLA122F TGLA122R TGLA122M13	CCCTCCTCCAGGTAAATCAGC AATCACATGGCAAATAAGTACATAC CACGACGTTGTAAAACGACCCCTCCTCCAGGTAAATCAGC	55

a The codes for each locus on the genetic map of bovine genome are put in parentheses. Source: FAO (2011),

b Forward primer whose sequence is provided with a tail M13 (sequence of 19 base pairs) to its 5 ‘end

Microsatellites were amplified by Li-Cor polymerase chain reaction (PCR) in simplex. The PCR reactions for 12 markers such as BM2113, BM1818, ETH10, ETH225, ETH152, HEL1, HEL9, INRA037, INRA063, MM12, TGLA53 and TGLA122, were ­performed in a 15 μl reaction volume containing 2.0 μl of DNA template and 13 μl of total PCR mix. The mix composed of 1.6 μl of 10X PCR buffer, 1.6 μl of dNTPs (2.5 mM), 0.8 μl of MgCl_2_ (25 mM), 0.2 μl of FM13 primer (10 μM), 0.3 μl of R primer (10 μM), 0.1 μl of Qiagen Taq DNA polymerase (5 U/μl) and 0.3 μl of dye M13 (700). The amplifications were carried out in a thermal cycler (BIOMETRA^®^ TGradient, version 4.20 g, Model No.1912460, Whatman) using the following conditions: Initial denaturation at 94°C for 3 min, followed by 35 cycles of 30 s at 94°C, 30 s at annealing temperature of 50, 55 or 60°C (according to the microsatellite) and 45 s extension at 72°C, then final extension at 72°C for 8 min ended the reactions. Subsequently, the amplified products were mixed with desmilings 700 (fluorescent dyes varying between 71 and 367 bp according to amplified microsatellite) in simplex rearrangements and were resolved on 6.5% denaturing acrylamide-urea gels using a Li-Cor^®^ automated sequencer (DNA Analyzer Model 4300) following the manufacturer’s procedures. All gels were analyzed using SAGA^GT^ Generation 2.0 software.

### Within-breed genetic diversity determination

The genetic variability of microsatellite loci and populations was measured by estimating a set of characteristic parameters of genetic polymorphism. Before the estimation of these measures, the presence of null allele across loci was checked using the program Micro-Checker version 2.2.3 [18]. Allele frequencies, observed number of alleles per locus (Na), observed heterozygosity (H_O_), unbiased expected heterozygosity (H_E_) [19], gene diversity of Nei (H_S_) [20], F_IS_ (*f*) (amount of inbreeding within a population of Weir and Cockerham [21]) were estimated using Genetix version 4.05.2 [22] and Fstat version 2.9.3.2 [23]. The significance test of values of the fixation index (F_IS_) was tested using methods of jackknifing and bootstrapping over loci after 1000 ­permutations of alleles within a population. The allelic richness of a breed is the measure of the number of allele’s independent of sample size which is estimated per locus (Rt) and population (Rs) using Fstat version 2.9.3.2 [23]. The principle of “Rarefaction” of Hurlbert (1971) suggested by El-Mousadik and Petit [24] was applied to correct the observed number of alleles according to the sample size. Other parameters such as the number of private alleles (NPA, alleles found in a single breed), effective number of alleles (Ne), Shannon’s information index (I) were determined using GenAlEx version 6.5 [25]. The polymorphic information content (PIC, a measure of informativeness of a marker, calculated according to Botstein *et al*. [26]) was estimated using Cervus 3.0.6, Field Genetics Ltd. [27]. The significant differences of Shannon’s Information index (I) and PIC between breeds were tested using *t*-test implemented in STATVIEW version 5.0 [28] at a significance level of 5%.

Exact tests for deviations from the Hardy-Weinberg equilibrium (HWE) were performed for each locus, in each population and for all populations using a Markov Chain Monte Carlo simulation (20 batches, 1,000,000 iterations per batch and a dememorization number of 10,000) implemented in Genepop 4 version 4.2.2 [29]. The significance of probabilities for all loci and populations was determined using Fisher’s method.

Test of the genotypic linkage disequilibrium was estimated between all pairs of loci using a G statistic (log - likelihood ratio) implemented in Fstat version 2.9.3.2 [23] to test the significance of association between genotypes at pairs of loci in each sample. The p-values of genotypic disequilibrium were based on 550,000 permutations. Adjusted p-value for 5%, 1% and 0.1% nominal levels was 0.000091, 0.000018, and 0.000002, respectively.

### Genetic distances and relationships among the populations

Genetic relationships among breeds were explored by multivariate statistical analysis and phylogenetic reconstruction. Genetic distances of Nei *et al*. [30] D_A_ were calculated through alleles frequencies to determine the genetic relationships among breeds using Genetix version 4.05.2. Moreover, an unbiased standard genetic distance of Nei [19] (D_S_), was calculated using the GenAlEx software version 6.5.

To condense the genetic variation revealed for the panel of 11 microsatellites loci, a multivariate analysis of microsatellite allele frequencies principal components analysis (PCA) was performed from the covariance matrix D_S_ using the GenAlEx program version 6.5.

Phylogenetic trees were generated using genetic distances that are suitable for numeric data. For this, two dendrograms of populations were constructed first from the distance matrix of Nei *et al*. [30] (D_A_) using the unweighted pair group method with arithmetic mean (UPGMA) [31] and the second from the chord distance (D_C_) of Cavalli-Sforza and Edwards [32] using the Neighbor-Joining (NJ) method of Nei [20]. We use genotypes of *Syncerus caffer* (African buffalo) from eight microsatellite data (ETH10, ETH152, ETH225, HEL1, HEL9, INRA037, INRA063, and TGLA53) [33] to root population trees. The construction was performed using Populations version 1.2.28 [34]. Dendrograms were visualized using Fig Tree version 1.4.2 [35].

## Results

### Within population genetic diversity

The genetic parameters per locus are shown in Table-2. Over the 12 microsatellite markers, 11 were found to be polymorphic at 100% in all populations with a total of 115 alleles detected. The average number of alleles was 10.45 per locus. The observed number of alleles per locus (Na) varied from 6 in INRA063 to 16 in TGLA53. Whereas, the allelic richness per locus (Rt) varied from 3.74 in INRA063 to 8.20 in TGLA53 with an average mean of 6.08. All markers showed high levels of heterozygosity (>0.60), except for INRA063, which generated H_O_ and H_E_ values of 0.44 and 0.60, respectively. All genetic makers showed PIC values higher than 0.5 with an average value of 0.76. Three of all loci (ETH225, HEL9, and INRA037) presented a positive value of F_IS_ overall populations, which was significantly different from zero (Table-2). So, the overall mean of inbreeding within populations (F_IS_) was 0.073 which implied a significant deficit of heterozygotes (p<0.05). The values of Chi-square with their p-values of BM2113 (χ^2^=24.9024, p<0.01), ETH152 (χ^2^=29.2500, p<0.001), INRA063 (χ^2^=19.9543, p<0.5) and INRA037 (χ^2^=15.2741, p<0.05) showed that these loci deviated very significantly from HWE in all populations. The test of linkage disequilibrium between different combinations of loci considering all samples showed none significant deviations.

**Table-2:** Genetic parameters measured per microsatellite locus.

Locus	Allelic range (bp)	N	Na	Rt	H_E_	H_O_	F_IS_ (WC)	PIC	HWE

									Chi-square value
BM1818	274-292	89	10	6.482	0.840	0.876	−0.049	0.815	3.2851^NS^
BM2113	140-164	85	9	6.578	0.847	0.671	0.194	0.823	24.9024**
ETH10	225-241	94	8	5.887	0.808	0.670	0.141	0.778	10.9206^NS^
ETH152	198-224	96	9	5.247	0.775	0.708	−0.020	0.737	29.2500***
ETH225	158-176	89	7	5.005	0.739	0.640	0.103*	0.699	9.5284^NS^
HEL1	121-141	102	11	6.369	0.844	0.814	0.023	0.820	8.9110^NS^
HEL9	164-190	102	14	7.318	0.870	0.804	0.063*	0.851	11.6563^NS^
INRA063	194-206	92	6	3.745	0.608	0.446	0.212	0.551	19.9543*
INRA037	132-154	74	12	5.750	0.802	0.716	0.048*	0.769	15.2741*
MM12	119-157	90	13	6.381	0.781	0.778	−0.014	0.750	4.9452^NS^
TGLA53	172-204	58	16	8.204	0.866	0.724	0.136	0.847	13.0689^NS^
Mean		88.27	10.455	6.088	0.798	0.713	0.073*	0.767	151.6963***

Parameters estimated per microsatellite locus across four Senegalese cattle breeds. N=Number of individuals typed per locus, Na=Observed number of alleles, Rt=Allelic richness, H_E_=Unbiased expected heterozygosity, H_O_=Observed heterozygosity, F_IS_ (*f*)=Amount of inbreeding within population computed following Weir and Cockerham, 1984, PIC=Polymorphic information content, χ^2^HWE=Chi-square values of test for HWE, NS: p>0.05=Not significant,

* p<0.05=Significant,

** p<0.01=Very significant,

*** p<0.001=Highly significant

**Figure-2:**
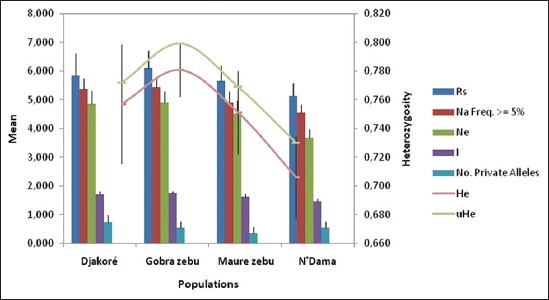
Mean allelic patterns across populations. Parameters estimated using 11 microsatellites in four Senegalese local breeds. Rs (mean allelic richness per locus); Na (frequency ≥5%) (Mean observed number of alleles with a frequency ≥5%/locus); Ne = (mean effective number of alleles/locus); I (Shannon’s information index); No. Private alleles (mean number of unique alleles to a single population); He (mean expected heterozygosity); uHe (mean unbiased expected heterozygosity).

The checking of null allele revealed that BM2113, INRA063 showed the evidence of null allele in Gobra zebu and HEL1 in Maure zebu. The genetic variability within a breed is resumed in Table-3 and Figure-2. The mean number of individuals typed per population (N) varied from 17±1.35 in N’Dama to 26.36±0.81 in Djakoré with an average of 22.06±0.74. The mean observed a number of alleles per population (Na=7.45±0.31) and the mean effective number of alleles per population (Ne=4.48±0.21) further confirmed the genetic variation in these four cattle breeds. Thus, the effective number of alleles was about 50% of the observed number of alleles. The mean NPA per population or the proportion of rare alleles within population ranged from 0.36±0.20 in Maure zebu to 0.72±0.27 in Djakoré with an average of 0.54±0.07. As regards to the mean allelic richness per population corrected for the sample size of each breed (R_S_), it ranged from 5.14 (N’Dama) to 6.10 (Gobra) (Figure-2). Therefore, the average gene diversity (H_S_) varied from 0.73 in N’Dama to 0.80 in Gobra (Supplement Table-2). The Gobra zebu had the highest value of PIC (0.75), and the lowest value was found in N’Dama (0.66). These differences showed that the population of Gobra zebu presented the highest within breed genetic variability. Regarding the Shannon’s information index (I), all cattle breeds presented a value distant from zero with an overall mean of 1.63±0.05. In addition, significant differences of this index were found among breeds.

**Table-3:** Genetic variability within cattle populations.

Cattle breeds	n	N (±SE)	Na (±SE)	H_E_ (±SD)	H_O_ (±SD)	F_IS_ (WC)	χ^2^HWE	PIC
Djakoré	30	26.36±0.81	8.091±0.78	0.772±0.140	0.752±0.188	0.026^NS^	47.9838**	0.728^a^
Gobra zebu	30	22.09±0.95	8±0.603	0.799±0.062	0.719±0.134	0.102*	34.2489*	0.752^b^
Maure zebu	30	22.81±1.26	7.364±0.544	0.769±0.099	0.725±0.113	0.059*	36.2507*	0.719^c^
N’Dama	30	17±1.35	6.364±0.453	0.730±0.106	0.643±0.152	0.123*	33.2128*	0.667^d^
Over all	120	22.06±0.74	7.455±0.311	0.768±0.047	0.710±0.047	0.073±0.026*	151.6963***	0.716±0.036

Parameters estimated using 11 microsatellites in four Senegalese local breeds. n=Number of individuals sampled/population, N=Mean number of individuals typed/population, Na=Mean observed number of alleles/locus, H_E_=Mean unbiased expected heterozygosity, H_O_=Mean observed heterozygosity, F_IS_ (*f*)=Within-population inbreeding coefficient and its confidence interval, computed following Weir and Cockerham, 1984, PIC=Polymorphic information content, χ^2^HWE=Chi-square values of test for HWE, NS: p>0.05=Not significant,

* p<0.05=Significant,

** p<0.01=Very significant,

*** p<0.001=Highly significant.

SE=Standard error, SD=Standard deviation,

a,b,c,d =Means of PIC in the same column followed by different letters are significantly different (p<0.05).

**Supplement Table-2:** Summary statistics of within breed genetic diversity.

Locus	Djakoré									Gobra zebu								
	
	N	Na	Rs	H_O_	H_E_	F_IS_	PIC	H_S_	PHWE	N	Na	Rs	H_O_	H_E_	F_IS_	PIC	H_S_	PHWE
BM1818	27	10	6.756	0.926	0.854	−0.086	0.818	0.853	NS	24	8	6.245	0.833	0.833	0	0.792	0.833	NS
BM2113	25	8	6.165	0.760	0.815	0.068	0.772	0.816	NS	24	8	6.653	0.542	0.858	**0.373**	0.820	0.865	S**
ETH10	25	8	6.254	0.840	0.829	−0.013	0.788	0.829	NS	22	7	5.450	0.591	0.773	**0.239**	0.721	0.777	NS
ETH152	26	8	6.077	0.885	0.835	−0.060	0.794	0.834	S***	26	6	4.908	0.769	0.742	−0.037	0.689	0.742	NS
ETH225	28	6	4.780	0.643	0.722	0.111	0.669	0.724	NS	22	6	4.611	0.682	0.708	0.038	0.652	0.709	NS
HEL1	29	7	5.871	0.897	0.831	−0.080	0.791	0.829	NS	25	10	7.314	0.880	0.873	−0.008	0.839	0.873	NS
HEL9	30	11	7.182	0.800	0.871	0.082	0.840	0.872	NS	25	11	7.773	0.840	0.870	0.035	0.838	0.871	NS
INRA063	28	3	2.620	0.250	0.382	**0.350**	0.334	0.385	S*	19	5	4.330	0.474	0.700	**0.329**	0.634	0.706	NS
INRA037	23	6	4.237	0.783	0.694	−0.131	0.623	0.692	NS	17	7	5.719	0.706	0.802	0.123	0.748	0.805	NS
MM12	28	12	7.539	0.821	0.840	0.033	0.816	0.850	NS	22	10	6.781	0.773	0.796	0.029	0.754	0.797	NS
TGLA53	21	10	6.777	0.667	0.810	**0.180**	0.768	0.813	S*	17	10	7.396	0.824	0.836	0.015	0.792	0.836	NS
Mean	26.36	8.091	5.842	0.752	0.771	0.026	0.728	0.772	S**	22.09	8	6.107	0.719	0.799	**0.102**	0.752	0.801	S*

**Locus**	**Maure zebu**									**N’Dama**								
	
	**N**	**Na**	**Rs**	**H_O_**	**H_E_**	**PIC**	**F_IS_**	**H_S_**	**PHWE**	**N**	**Na**	**Rs**	**H_O_**	**H_E_**	**PIC**	**F_IS_**	**H_S_**	**PHWE**

BM1818	22	7	5.894	0.864	0.831	0.785	−0.040	0.830	NS	16	8	5.968	0.875	0.821	0.765	−0.068	0.819	NS
BM2113	22	9	6.410	0.727	0.818	0.775	0.113	0.820	NS	14	6	5.434	0.643	0.815	0.753	0.217	0.821	NS
ETH10	26	7	5.311	0.654	0.784	0.734	0.168	0.786	NS	21	7	4.931	0.571	0.713	0.646	**0.202**	0.717	NS
ETH152	24	7	4.256	0.750	0.640	0.578	−0.176	0.638	NS	20	4	3.546	0.350	0.517	0.466	0.328	0.521	S*
ETH225	25	5	4.203	0.560	0.665	0.607	0.161	0.668	NS	14	7	5.783	0.714	0.783	0.722	0.090	0.786	NS
HEL1	28	7	6.075	0.643	0.842	0.804	**0.240**	0.846	S*	20	8	5.483	0.850	0.771	0.714	−0.106	0.768	NS
HEL9	26	9	7.062	0.883	0.867	0.833	−0.020	0.867	NS	21	8	6.422	0.667	0.867	0.765	**0.177**	0.811	NS
INRA063	22	4	3.682	0.591	0.580	0.523	−0.018	0.580	NS	23	5	3.657	0.522	0.655	0.576	**0.207**	0.658	NS
INRA037	21	7	5.684	0.762	0.811	0.761	0.061	0.812	NS	13	7	5.647	0.538	0.689	0.634	0.225	0.696	S*
MM12	23	10	5.960	0.870	0.744	0.694	−0.173	0.741	NS	17	4	3.694	0.588	0.626	0.554	0.061	0.627	NS
TGLA53	12	9	7.688	0.667	0.877	0.821	0.247	0.886	NS	8	6	6	0.750	0.833	0.748	0.106	0.839	NS
Mean	22.81	7.364	5.657	0.724	0.769	0.719	**0.059**	0.770	S*	17	6.364	5.142	0.642	0.735	0.667	**0.123**	0.733	S*

Number of individuals typed per locus (N); observed number of alleles per locus (Na); allelic richness per locus (Rs); observed (H_O_) and unbiased expected heterozygosity (H_E_); gene diversity of [20] (HS); amount of inbreeding within populations according to Weir and Cockerham, 1984 (FIS). PIC=Polymorphic information content; p values of test for HWE (PHWE); permutation tests (1000 replicates) of the inbreeding coefficient (FIS): Values in bold correspond to significant tests (percentage of replicates with a value of FIS less than that observed, i.e., >95%); NS: p>0.05=Not significant;

* : p<0.05=Significant,

** p<0.01=Very significant,

*** p<0.001=Highly significant

The overall mean values of observed heterozygosity (0.71±0.04) and expected heterozygosity (0.76±0.04) indicated the presence of high level of heterozygosity in native local cattle breeds. The F_IS_ values indicated that three breeds (Gobra zebu, Maure zebu, and N’Dama) presented a significant deficit of heterozygotes (p<0.05). Considering all populations and all loci, a highly significant deviation from HWE was noted (χ^2^=151.6963, p<0.001).

### Genetic distances and breed relationships

**Figure-3:**
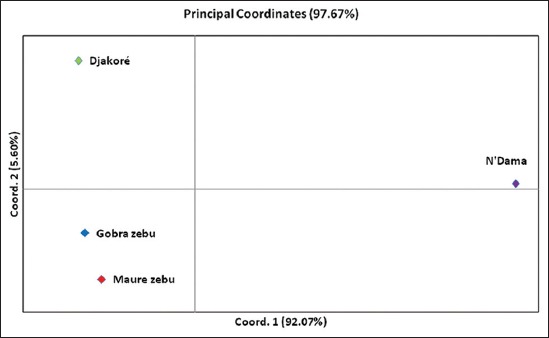
Diagram of principal coordinates analysis based on covariance matrix of Nei’s unbiased genetic distance.

**Figure-4:**
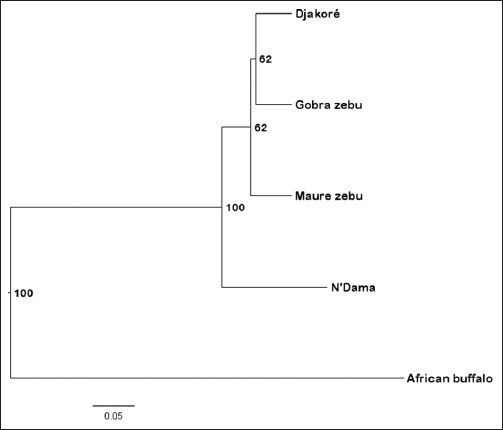
Phylogenetic tree constructed from D_A_ [30] by the unweighted pair group method with arithmetic mean method showing genetic relationships among four Senegalese cattle breeds. Numbers represent the percentage of times that a node occurred in 10,000 bootstrap replicates. The linear scale relates the branch lengths to units of D_A_. The root of the tree was placed at the midpoint of the longest branch separating the African buffalo from the other groups.

**Figure-5:**
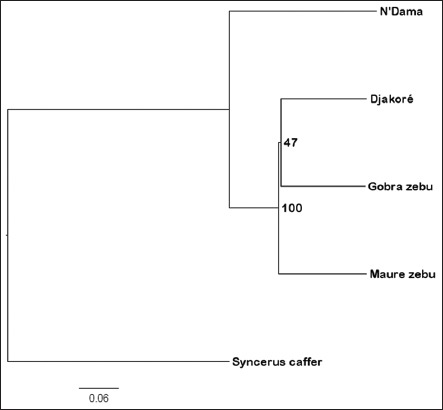
Neighbor-Joining tree showing genetic relationships among four Senegalese cattle breeds using D_C_ genetic distances [32]. The numbers on the nodes are percentage bootstrap values for 10,000 replications. The linear scale relates the branch lengths to units of D_C_. The root of the tree was placed at the midpoint of the longest branch separating the *Syncerus caffer* from the other groups.

Allele frequencies were used to calculate Nei’s unbiased genetic distances (D_S_) and Nei D_A_ genetic distances for each pair of the four cattle populations (Table-4). As regards to both genetic distances, the N’Dama appeared as genetically more remote from the other breeds. As expected, the N’Dama is a taurine breed. By the D_S_ genetic distance, the Gobra zebu and Maure zebu as genetically the closest populations; whereas recording to values of D_A_, Djakoré, and Gobra zebu are the most related populations (Table-4). So regarding the low values of genetic distances between the Gobra zebu, Maure zebu, and Djakoré, these three breeds share closest genetic similarities. PCA was performed, including all populations and loci using the covariance matrix of Nei unbiased genetic distance (D_S_) to summarize breed relationships (Figure-3). A total of 97.67% of the variance accounted for the first two dimensions of the PCA (Figure-3). The first principal components (PC) that accounts 92.07% of the total genetic variability distinguished clearly the N’Dama to the remaining populations. The second PC, which summarizes 5.60% of the variation, separated evidently Djakoré breed to Gobra and Maure zebu’s populations. Therefore, in the multivariate space defined by the two first PCs, the zebu populations are grouped together as genetically identical populations. Visualization of breed relationships was done further by constructing different trees. So, both rooted UPGMA and NJ dendrograms by a related species known as *S. caffer* (African buffalo), have revealed that cattle populations are distinguished strongly into two major clades (Figures-4 and 5). The N’Dama was the most distinct and separated first. The second clade clustered the remaining populations as Djakoré, Gobra zebu, and Maure zebu with more than 60% bootstrap value in trees. This showed that these three populations had the same genetic ancestry, which reflects their strong phylogenetic relationships shared. The subclade formed by the Djakoré and Gobra breed in both UPGMA and NJ trees with a percentage of bootstrap of 62% and 47% respectively, showed that the Djakoré is genetically more apparent with the Gobra than with the N’Dama.

**Table-4:** Pairwise population genetic distance values among four Senegalese cattle breeds.

Breeds	Djakoré	Gobra zebu	Maure zebu	N’Dama
Djakoré	-	0.029	0.038	0.460
Gobra zebu	0.036	-	0.018	0.442
Maure zebu	0.041	0.042	-	0.412
N’Dama	0.107	0.102	0.104	-

Nei D_S_ unbiased distances [19] are shown above diagonal and Nei D_A_ distances [30] are shown below diagonal

## Discussion

Genetic diversity, along with phylogenetic relationships, was examined using microsatellite markers within and among the four local cattle breeds from three agro-ecological areas of Senegal.

### Genetic variability within populations

Since the biotechnology of artificial insemination and changes of production systems have become widespread in the main cattle breeding areas during the past two decades, few reports have comprehensively detailed the genetic diversity of currently important Senegalese local breeds described in this study. While, the most recent data back to studies of MacHugh *et al*. [15] and Freeman *et al*. [17].

Most of the loci used in this work had been analyzed in previous studies with different breeds such as European cattle breeds, Near East cattle breeds, African taurine and zebu and Asian zebu [15,17,36,37]. However, the analysis of microsatellite polymorphisms revealed that the average number of alleles per locus obtained (Na=10.45) was in the same range of that reported in Cameroonian indigenous cattle (Na=10.69), in Togolese and Beninese cattle (Na=10) [38,39]. The mean PIC value (0.76) was as higher as the 0.75 generated in Cameroonian cattle breeds [39] so very informative. Moreover, the average values of allelic richness and heterozygosity showed that these loci give reliable information on genetic diversity and population structure of breeds.

The four Senegalese cattle breeds and particularly the putative zebu populations displayed considerable levels of genetic diversity as estimated by allelic richness (Rs), expected heterozygosity (H_E_) and gene diversity (H_S_). The mean allelic richness (Rs) varied from 5.1 in N’Dama to 6.1 in Gobra. MacHugh *et al*. [15] had found a value of Rs of 4.5 in N’Dama, 4.8 in Gobra zebu and 5.3 in Maure zebu. This trend was confirmed by assertions of Freeman *et al*. [17] where the breeds located proximal to the perimeter of the tsetse zone (e.g. Maure, Gobra, Kuri, Peul Fulani and Borgou) tend to display highest values of allelic diversity than most other resident breeds within this area. Moreover, the level of the allelic richness found in Borgou (“hybrid” zebu × taurine) within West and Central African cattle [40] is similar to that found in Djakoré. Indeed, “hybrid” populations tend to have a high value of Rs. This suggests that a large allelic richness may reflect the “heterogeneity” of the breed. Thought, the Djakoré cattle are supposed as a “hybrid” population by phenotypic characteristics and its geographic distribution [4]; crossbreeding factors has led it to become a newly stabilized breed. Thus, according to Ndiaye *et al*. [41], characters which the Djakoré has inherited from his both parents have allowed its adaptation in its own production system. For a more general point of view, reproductive isolation between a homoploid “hybrid” species and its parents is generally attained by chromosomal rearrangements, ecological divergence, and/or spatial isolation from the parental species. These factors prevent the incipient “hybrid” species from being genetically swamped through mating with the parental species, and allow it to evolve as an independent lineage [42]. In this case, ecological divergence may be compared to a particular livestock production system where the Djakoré is bred. Moreover, African zebu breeds have been influenced by historical zebu-taurine crossbreeding and the high allelic diversity observed is undoubtedly an artifact of admixture and the consequent input of both taurine and zebu alleles [15]. As consequent, levels of allelic diversity can evolve during the time. Foulley and Ollivier [40] confirmed this pattern of evolution in the case where many geneticists have underlined the importance of the number of alleles in a perspective of genetic amelioration of long-term since there is a link between allelic richness and evolutionary history of populations.

Senegalese cattle breeds showed, in general, high proportion of rare alleles with an average mean of 54%. This showed that the specificity of the variability generated by each population is due to state of certain alleles which are own. The typical case is the Djakoré population which had the highest mean NPA (0.72). Similar results were obtained in Borgou cattle which possessed the highest number of rare alleles among West African and Central zebu and taurine breeds [40]. Within the 24 rare alleles detected amongst the 4 breeds, only 3 had a frequency higher than 5%, this is the case of *INRA063-206 bp* with a frequency of 5,3% in Gobra zebu, *INRA037-134 bp* and *TGLA53-174 bp* which reached a frequency of 11,5% and 6,3%, respectively in N’Dama (Supplement Table-3). We specify that allele’s size is augmented by 19 bp. Since on the Licor, a primer FM13 with a M13 tail of 19 bp was used. Contrary, results of MacHugh *et al*. [15] have found all private alleles detected in one breed with a frequency lower than 5%. Regarding the average Shannon’s information index (1.63), it can be avowed that Senegalese local breeds have considerable genetic variability. In addition, the significant differences observed revealed that the Gobra had the highest within population variability.

**Supplement Table-3:** List of private alleles with frequency across loci per cattle breed.

Cattle breeds	Locus	Alleles	Frequency
Djakoré	BM1818	288	0.019
		292	0.019
	ETH152	204	0.019
		224	0.019
	INRA037	138	0.022
		140	0.022
	TGLA53	172	0.048
	HEL9	190	0.017
Gobra zebu	HEL1	137	0.020
		141	0.040
	INRA063	206	0.053*
	INRA037	154	0.029
	MM12	153	0.023
	HEL9	180	0.040
Maure zebu	ETH152	222	0.021
	INRA037	136	0.024
	TGLA53	196	0.042
		204	0.042
N’Dama	ETH225	176	0.036
	HEL1	135	0.025
	INRA063	194	0.022
	INRA037	132	0.038
		134	0.115*
	TGLA53	174	0.063*

* Alleles with frequency>5%

The mean expected heterozygosity (H_E_) per breed varied between 0.73 for the N’Dama and 0.79 for the Gobra zebu. Our results showed a considerable level of heterozygosity among the four cattle breeds. A similar level of heterozygosity was reported in Togolese and Beninese, Mozambican and Cameroonian cattle zebu and taurine breeds [38,39,43]. As expected, the microsatellite loci showed very high level of gene diversity, with an average within population gene diversity (H_S_) ranged from 0.73 (N’Dama) to 0.80 (Gobra). The high values of allelic diversity, expected heterozygosity and gene diversity obtained in this study well confirm that Senegalese local cattle breeds represent an important reservoir of genetic variability and they reflect the absence of selection or organized breeding programs for Senegalese cattle, contrary to highly selected breeds which display lower diversity due small effective population sizes [44].

Levels of genes diversity were similar for all breeds, suggesting that there are no appreciable differences in the amount of genetic variability among Senegalese breeds. By comparing the level of genetic variation amongst the four Senegalese cattle breeds, this from the Upper Casamance area (N’Dama) displayed the lowest within breed variability. Since the N’Dama breed is reared in an isolated breeding area separated by the “Gambia River” from the other cattle production systems, it must be less affected by intensive uncontrolled crossings. Therefore, according to investigations study, none of the herds of Gobra, Maure and Djakoré breeds practiced transhumance toward the Upper Casamance area; and 44% of transhumant N’Dama herds, did not exceed a range of 30 km out of the Kolda Region. Furthermore, the amount of genetic diversity in these breeds was comparable to those reported for other cattle breeds in different regions of Africa [15,17,38,39,43]. These high diversity indices that harbor the current Senegalese cattle breeds can be explained mainly by the presence of genes from two genetically differentiated groups namely taurine and zebu. So, using the cytochrome B gene, Ndiaye *et al*. [12] found also a high genetic variability among local and exotic cattle reared in Senegal. Thus, it could be due so to intensive inbreeding occurring within the breeding tract of these local cattle.

A significant deficit of heterozygosity (p<0.05) was found in Gobra, Maure and N’Dama breeds. Thus all populations has deviated from HWE. A considerable variance of the deficit (F_IS_) between subpopulations might due mainly by population substructure as regards to the strong difference on null allele frequency across loci under a high level of genetic differentiation [45]. Hence, we could exclude the influence of null alleles on heterozygosity deficiency observed in our populations as the loci who have presented the signs of null alleles in two populations are different to those showed a deficit of heterozygotes. However, our results differed from those of MacHugh *et al*. [15] where only Maure zebu gave a significant deviation at the p<0.01 level. This deviation was due to site, period, and size of sampling of individuals Maure zebu breed. This deficiency of heterozygotes among populations is an indicator of inbreeding among cattle breeds or the occurrence of population substructure. Here, Hardy-Weinberg disequilibrium might be attributed to population subdivision owing to sampling of each breed was done from a range of distinct locations within the same broad geographical area when panmixia is unlikely to occur.

### Relationships among the breeds

All the genetic distance measures employed to estimate inter-breeds closeness showed, in general, low genetic divergence between the four cattle breeds. Belonging to taurine subspecies, the N’Dama remained the most genetically divergent population, while the Gobra, Maure zebu, and Djakoré are closer related populations. These values of genetic distances observed among Senegalese cattle were comparable to those obtained among West and Central African zebu and taurine cattle [17], Mozambican cattle [43] and Cameroonian cattle breeds [39]. Phylogenetic analysis described the same relationships shared by the four cattle breeds that the genetic distances. Therefore, the finest phylogenetic relationship was found between Djakoré and Gobra. This showed that the Djakoré shared more identical alleles with Gobra than with N’Dama. In effect, the same relations between Djakoré and Gobra zebu were reported by discriminant factor analysis using phenotypic characters [41]. And 5.88% of the Gobra cattle were classified in Djakoré cattle, whereas, the percentage of well-classified animals was 100% in Djakoré cattle [41]. This might be due to the consequence of the zebu gradient introgression which showed that the gene pool of Djakoré population is largely constituted by the Gobra zebu genome. In addition, according to MacHugh *et al*. [15], the distribution of zebu alleles and the zebu admixture proportions declines from East to West Africa and then follow a steep north-south gradient in West Africa. Moreover, the genetic relationships of these four cattle breeds correspond to their breeding history and geographic origins. Effectively, where the level of *Bos indicus* admixture in the trypanotolerant N’Dama populations is almost certainly the result of selection against introgressing breeds in the humid tsetse regions of West Africa [17]. These phylogenetic relationships found in Senegalese cattle were similar to those reported by Freeman *et al*. [17] and Bessa *et al*. [43] where phylogenetic relationships including European taurine, Indian zebu, African taurine, African zebu, and West African “hybrids” breeds were explored.

However, in phylogenetic methods, it is very difficult to separate the effect of admixture from that a common ancestry. Multivariate analysis of microsatellites allele frequencies has been a powerful tool to reveal underlying evolutionary history and admixture among distantly populations [46]. Hence, the grouping pattern of PCoA revealed a great genetic admixture between the zebuine breeds (Gobra and Maure). This grouping of Gobra and Maure was expected because according to Ndiaye *et al*. [41], 75% of Gobra-Maure herds practiced seasonal transhumance and crossed during other herds of the same breeds. This proves that Gobra and Maure cattle mate often between them without any control. Therewith, we can supposed that the genetic mixtures occurred most between Gobra and Maure zebu populations than the other pairs of breeds owing to that there were reared in the same production environment. Furthermore, the same multivariate space shared by the Djakoré cattle with the zebu populations demonstrates clearly that it belongs to *B. indicus* subspecies. As confirmed by genetic distances, the PCs distinguished clearly the N’Dama breed to the other cattle populations. Therefore, Freeman *et al*. [17] confirmed this divergence between *Bos taurus* and *B. indicus* using the PCA analysis including most West African cattle.

## Conclusions

This study based on polymorphism of microsatellite markers revealed that the Senegalese cattle breeds had a considerable level of genetic diversity. Therefore, hybridization, the major influence on allelic diversity in these populations, tends to increase diversity by bringing together alleles from the two distinct lineages (*B. taurus* and *B. indicus*). In addition, the high rate of inbreeding affecting these populations could destabilize the level of variability of Gobra and Maure zebu to the benefit of Djakoré population. If measures of rearing and conservation strategies are not promptly taken, the melting of genetic pools of different populations would lead to the disappearance of certain Senegalese local cattle breeds. Because, the loss of diversity linked to the disappearance of a breed is measured by the number of alleles that are specific. Priorities of conservation based on allelic diversity can be established.

This work is the first detailed study about the genetic variability and phylogenetic relationships of Senegalese cattle breeds. These breeds are important nutritional and economic resources for Senegalese people, and their high variability makes them suitable candidates for conservation and improvement to disconcert to global changes.

Conservation of genetic variability in these populations should be considered by breeders, in the interest of long-term future of the populations in their native tract. To begin with, breed societies/associations need to be created, that will be responsible for registration of these cattle populations as breeds, complete maintenance and improvement of the breed to make it economically sustainable in the transforming agricultural scenario of the country.

## Authors’ Contributions

MS and GJS conceived and supervised the entire study. NPN performed microsatellite genotyping under the supervision of GKD. NPN carried out genetic analysis, drafted and revised the manuscript under the guidance of AS and SN. All authors read and approved the final manuscript.
